# Endovascular treatment of spinal AVM: report of two cases with transvenous approach in combination with retrograde pressure cooker technique

**DOI:** 10.1007/s00234-023-03132-x

**Published:** 2023-03-02

**Authors:** Christian Paul Stracke, Wolfram Schwindt, Lukas Meyer, Jens Fiehler, René Chapot

**Affiliations:** 1 Clinic for Radiology, Section for Interventional Neuroradiology, University Hospital Münster, Albert-Schweitzer-Campus A1, 48149 Münster, Germany; 2 Department for Neuroradiological Diagnosis and Intervention, University Medical Center Hamburg-Eppendorf, Hamburg, Germany; 3 Department of Radiology and Neuroradiology, Alfried Krupp Krankenhaus, Essen, Germany

**Keywords:** Spinal AVM, Retrograde embolization, Ethylenvinylalcohol-polymer, Pressure cooker

## Abstract

**Purpose:**

Endovascular treatment of spinal AVMs is limited by low complete cure rates. Transarterial extensive treatment with liquid embolics carries the risk of clinically relevant ischemic complications. We report two cases of symptomatic spinal AVMs treated by a *transvenous* approach with retrograde pressure cooker technique.

**Methods:**

In two selected cases, transvenous navigation aimed at retrograde pressure cooker embolization.

**Results:**

Retrograde venous navigation was possible with two parallel microcatheters, and the pressure cooker technique with ethylenvinylalcohol-polymer was applicable in both cases. One AVM was occluded completely, and one subtotally due to a second draining vein. No clinical complications occurred.

**Conclusion:**

A transvenous approach for embolization with liquid embolics may offer advantages in treating certain spinal AVMs.

## Introduction

Spinal arteriovenous malformations (AVMs) are rare conditions that become clinically significant either by bleeding or progressive neurological symptoms [1, 2]. In a systematic review [3], the annual bleeding rate of glomus type II AVMs was 4%, increasing to 10% after the first hemorrhage. Rangel-Castilla [4] reported a large single-center series of 66 patients with spinal AVMs treated for 13 years with multimodal, predominantly surgical techniques. The clinical presentation consisted of bleeding in 37%, whereas most patients presented with paralysis or paresis.

Complete obliteration rates have been reported with 78% for surgery [3] and 33% for endovascular treatment. Long-term clinical worsening was noted, with 12% for surgical and 13% for endovascular treated patients. Since no large trials are available, the reports about treatment are limited to small case series with heterogeneous populations. Recently, stereotactic radiosurgery results were published [5], but the experience remains even more limited. Due to the vascular anatomic features and the highly functional spinal cord system, the potential risk of any treatment is high.

Endovascular therapy includes embolizations with glue (NBCA), particles, coils, or modern liquid embolic agents like ethylenvinylalcohol-polymer (Onyx®, Medtronic, Minneapolis, MN, USA; Squid®, Balt, Montmorency, France) [6–8]. However, transarterial embolization carries the risk of ischemic embolic complications that depend on the catheterization technique and the embolic agent [9, 10]. These limitations of endovascular therapy often lead to incomplete occlusion. Also, a palliative treatment concept with a long-term approach to treat risk structures like intranidal aneurysms is possible, or flow reduction to potentially prevent slowly progressive neurologic symptoms [11].

Recently, new endovascular techniques for intracranial AVMs evolved, such as the pressure cooker technique [12] and transvenous procedures [13–17]. These techniques reflect the intention to maximize control and minimize the risk of arterial ischemia. The anatomy of the draining veins of a spinal AVM is variable and often very tortuous, but in selected cases, the venous root can be accessible for transvenous catheterization. Here we report two transvenous treatments with coils, glue, and the polymeric liquid embolics Onyx and Squid.

## Case descriptions

### Patient #1

The 44-year-old female was diagnosed with a cervical spinal AVM in 1993. The AVM was partially embolized transarterially twice. In 2007 she presented with a SAH of the craniocervical junction. An intranidal aneurysm was likely to be the bleeding cause and was therefore embolized with coils. The patient recovered entirely from the SAH. During the subsequent years, three transarterial embolizations followed. Since 2007, the patient has had spastic palsy, mainly on the left side, with a sensorimotor deficit below Th1, a sensory palsy on the right side below Th3, and a bladder disturbance with urine retention of about 300 ml. During the following years, there was a slight deterioration of neurological symptoms. No further bleeding occurred. Control angiography in 2017 showed a residual supply of the AVM from both vertebral arteries. With the patient’s informed consent, we planned a definite endovascular treatment.

### Endovascular procedure

**Fig. 1:**
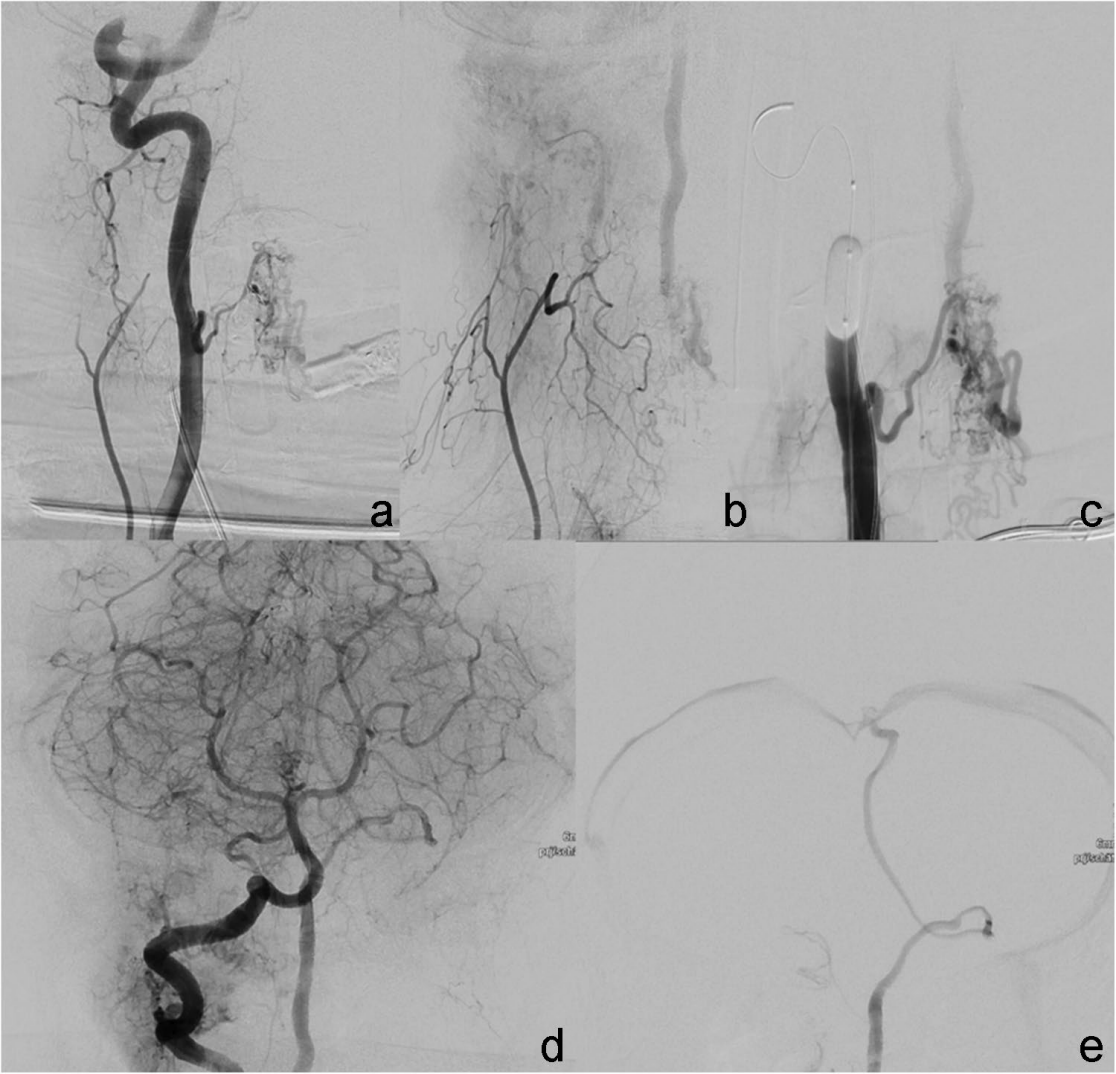
Patient 1: arterial supply of the spinal AVM remnant from the right vertebral artery via the anterior spinal artery system (**a**). Venous drainage, initially obscured by the overprojection of arteries of the posterior circulation (**b**, **c**), is clearly depicted (**d**, **e**) after transient vessel occlusion of the right vertebral artery by a remodeling balloon (**d**)

The angiography showed only a few small remaining arteriovenous shunts (Fig. 1a). The feeding arteries were supplied from arteries to the anterior spinal system from the right and left vertebral arteries. The draining vein showed a straight course upward (Fig. 1b). The exact intracranial course, however, could not be delineated due to the over-projection of the posterior intracranial circulation (Fig. 1c). Blocking the latter transiently by a remodeling balloon in the vertebral artery allowed the exact delineation of the course of the draining vein intracranially (Fig. 1d and e).

**Fig. 2:**
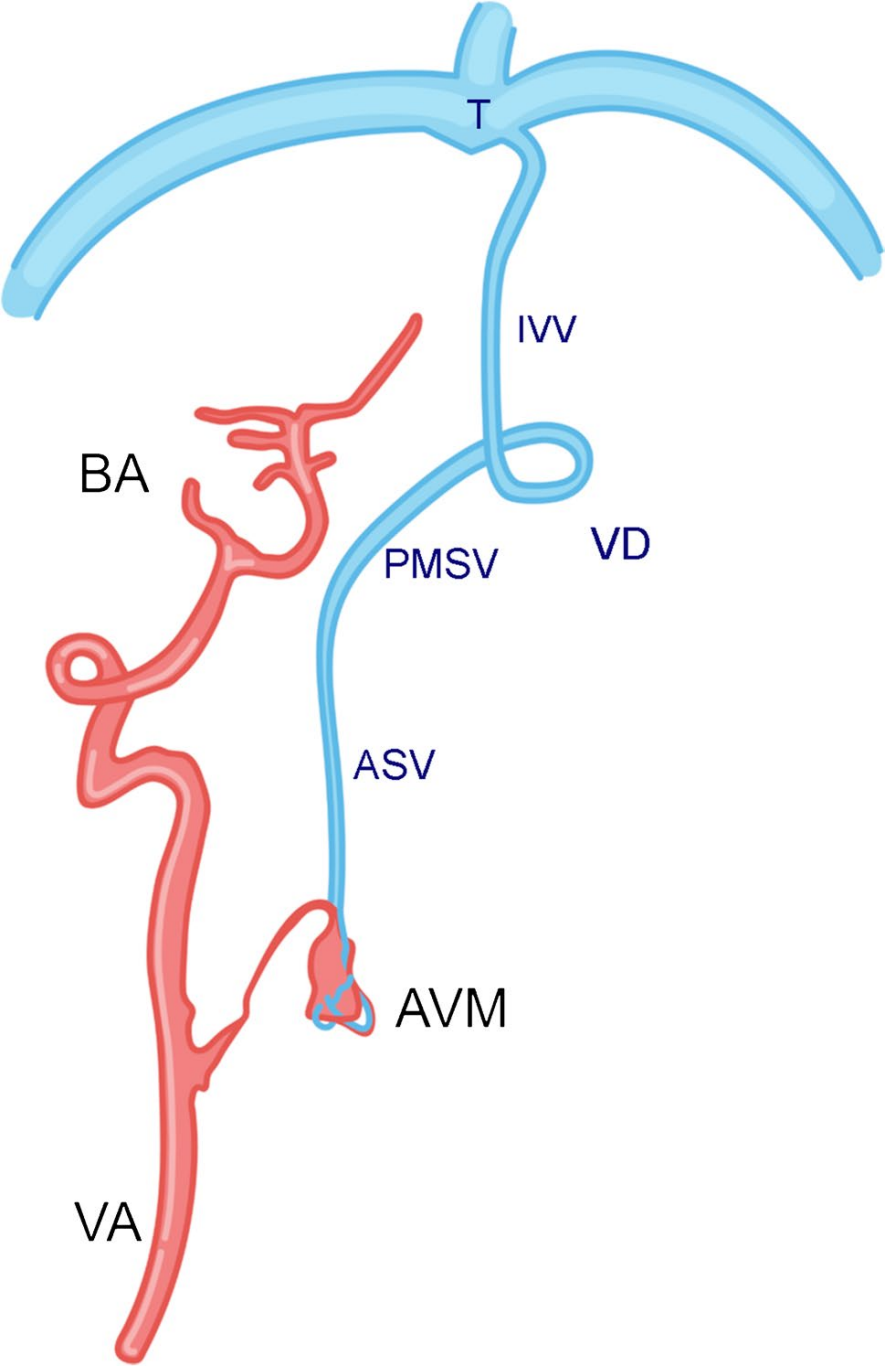
Patient 1. Sketch of pathologic vessel anatomy with the spinal AVM remnant (VA, vertebral artery; BA, basilar artery). Venous drainage via the anterior spinal vein (ASV), pontomedullary sulcus vein (PMSV), inferior vermian vein IVV, into the torcular (T)

For a better overview of vessel pathology, Fig. 2 sketches the main arterial supply of the AVM from the right vertebral artery and its spinal and intracranial venous drainage around the brain stem (pontomedullary sulcus vein) and via the inferior vermian vein into the torcular.

**Fig. 3:**
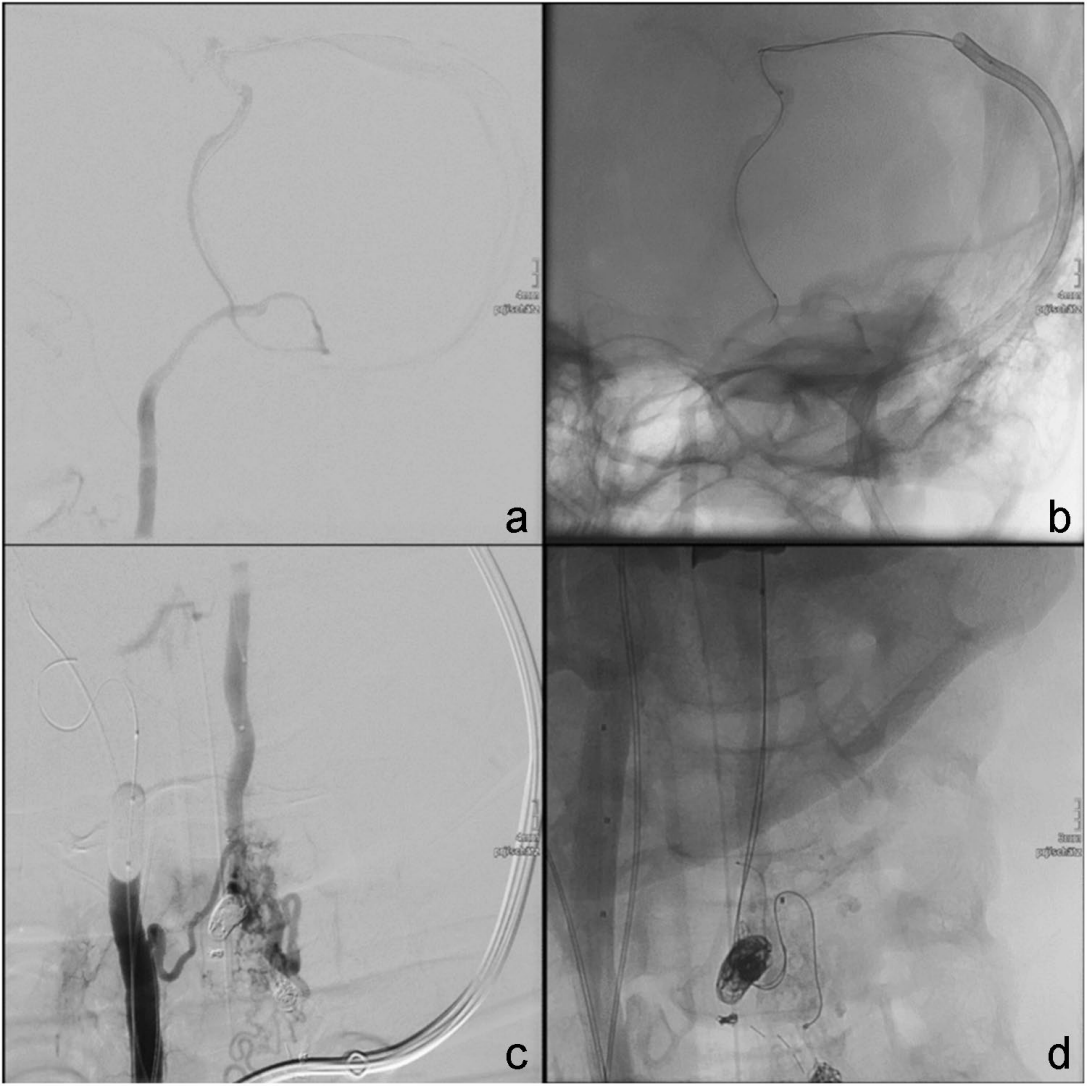
Patient 1: Transvenous navigation of microcatheters (Magic 1,2 FM, Sonic 1,2 F 25) via 7F guiding catheter in the transverse sinus (**a**–**c**) and transvenous embolization with Onyx 18 in pressure cooker technique (**d**)

Retrograde navigation started with inserting a 7 F sheath into the left jugular vein and a 7 F guiding catheter (Envoy, Johnson & Johnson, New Brunswick, NJ, USA) into the left transverse sinus in coaxial technique. Using a superselective roadmap achieved by transarterial injection into the right VA, the retrograde navigation of a Magic 1.2 FM microcatheter and a Sonic 1.2 F 25 detachable tip microcatheter was feasible using a Hybrid 007″ guidewire (BALT, Montmorency, France) (Fig. 3).

After exact anatomical delineation of the venous origin, embolization in pressure cooker technique [12] was performed: through the magic microcatheter, three injectable coils (Flow coil, BALT, Montmorency) were implanted, followed by the injection of NBCA (Histoacryl, B.Braun, Melsungen, Germany) combined with LIPIODOL® (Guerbet, Villepinte, France) in a 1:1 mixture. After retrieval of the Magic microcatheter, the embolization through the SONIC microcatheter was started with DMSO, followed by Onyx 18. The root of the primary draining vein was occluded completely. The embolization was stopped when Onyx appeared in the direction of the anterior spinal artery.

**Fig. 4:**
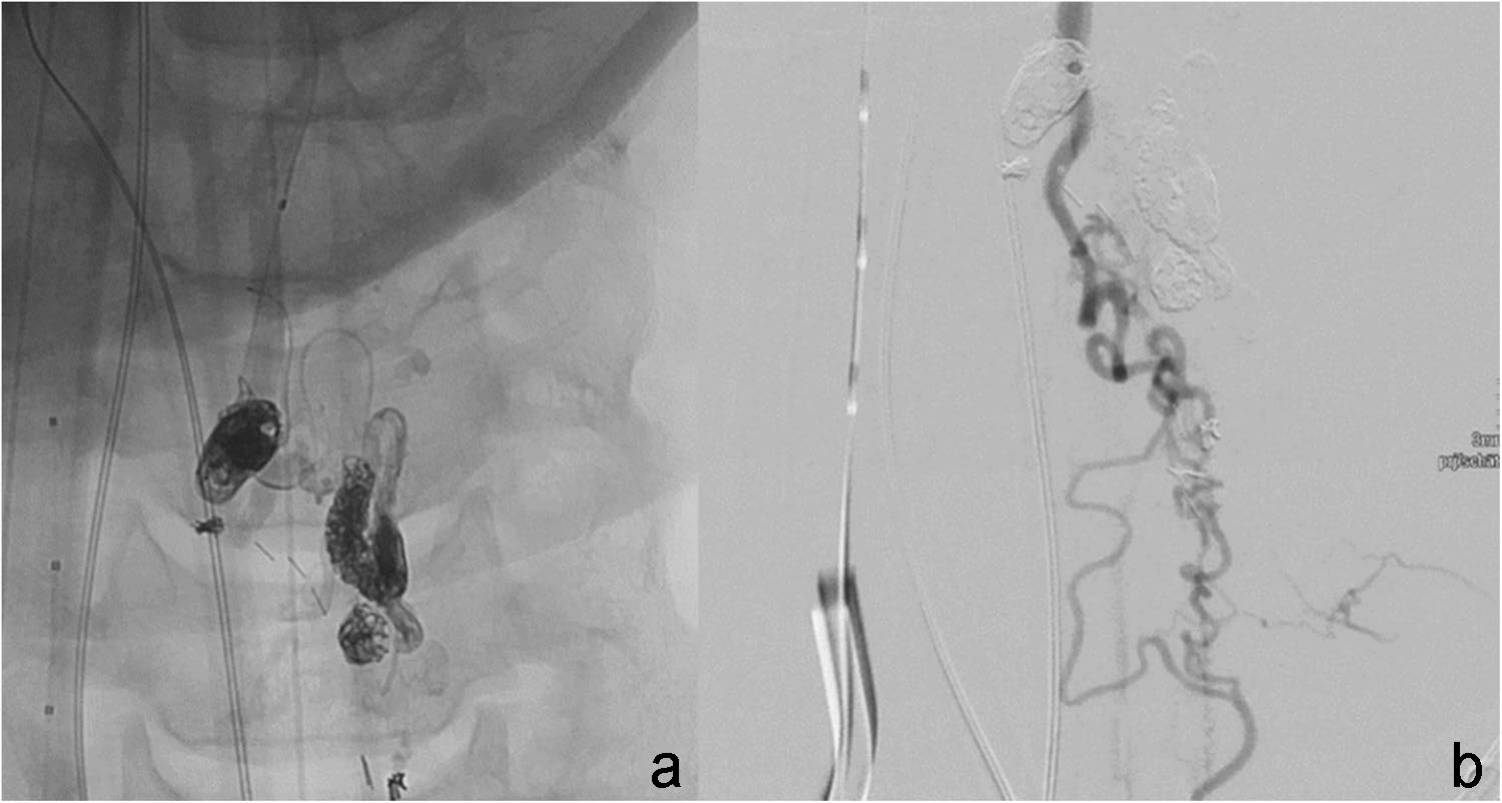
Patient 1. Retrograde pressure cooker embolization (**a**) with the final angiographic result (**b**) with arterio-arterial collateral flow without remaining shunts, complete occlusion of the treated vein, small remaining shunts from the left vertebral artery to a second draining vein, and patency of the large spinal vein

The retrieval of the catheter was possible with low force. Final angiographic controls showed complete occlusion of the treated vein and small residual shunts from the left vertebral artery to a second draining vein (Fig. 4). The large spinal vein remained patent.

### Clinical result and follow-up

The patient showed no new postprocedural neurological deficits and was discharged home on the third postoperative day. Up to now, the patient is neurologically stable, and no further treatment has been initiated.

### Patient 2

A 28 male patient presented with progressive paraparesis; symptoms had developed during 2 weeks. MR imaging showed severe edema of the lower thoracic spinal cord with multiple flow voids surrounding the spinal cord.

**Fig. 5:**
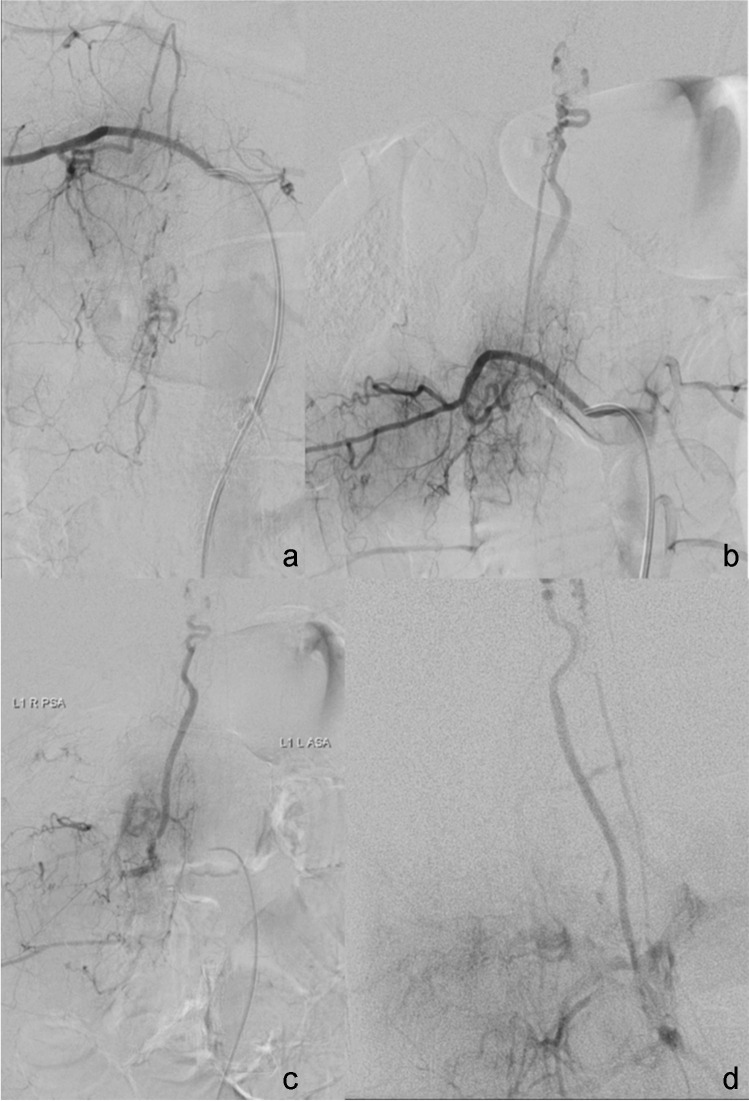
Patient 2. Spinal AVM Th11 with feeders from two radiculomedullary arteries supplying the posterior spinal artery originating from the right intersegmental arteries Th10 (**a**) and L1 (**b**), no feeders from two radiculomedullary arteries originating from the corresponding left arteries Th10 (**a**) and L1 (**c**) that supplied the anterior spinal artery. Venous drainage via right lumbal vein L1 into inferior vena cava, VCi (**d**). See also Fig. 6

**Fig. 6:**
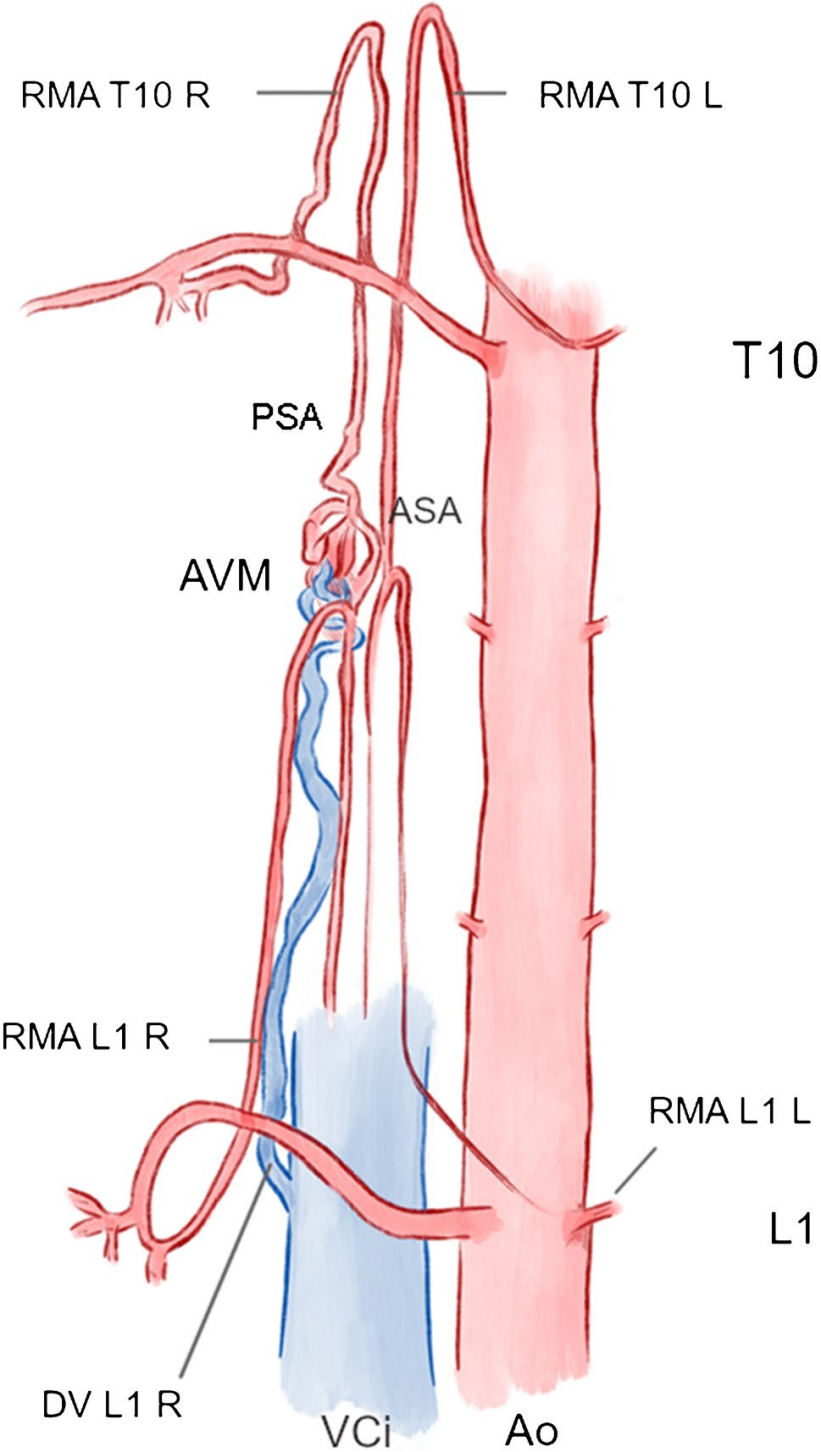
Patient 2, sketch of vascular pathology: thoracicolumbal spinal AVM supplied from radiculospinal arteries originating from the right intersegmental arteries Th10 and L1 supplying the posterior spinal artery. No feeders from the anterior spinal artery that are supplied by radiculomedullary arteries originating from the corresponding vessels (Th10, L1) on the left. Venous drainage into the inferior vena cava (VCi) via the right lumbal vein L1

Spinal angiography showed an AVM mainly supplied by the posterior spinal system with the main feeding arteries from the right 10th thoracic intersegmental artery (Th 10) and right first lumbar (L1) intersegmental artery. Two radiculomedullary arteries originated from Th10 and L1 left and supplied the anterior spinal artery but not the AVM (Fig. 5a–d, sketched in Fig. 6).

The nidal structure, moreover, showed aneurysmatic changes, with one aneurysm of more than 8 mm in size. The first endovascular treatment consisted of coil embolization of the large aneurysm followed by embolization with Onyx 18 via Th10 right and L1 right using two microcatheters.

During the following 4 months, the motoric deficits decreased. The patient was admitted again due to progressive pain in the right leg. MRI did not show any significant change in comparison to the imaging immediately after the first endovascular procedure.

**Fig. 7:**
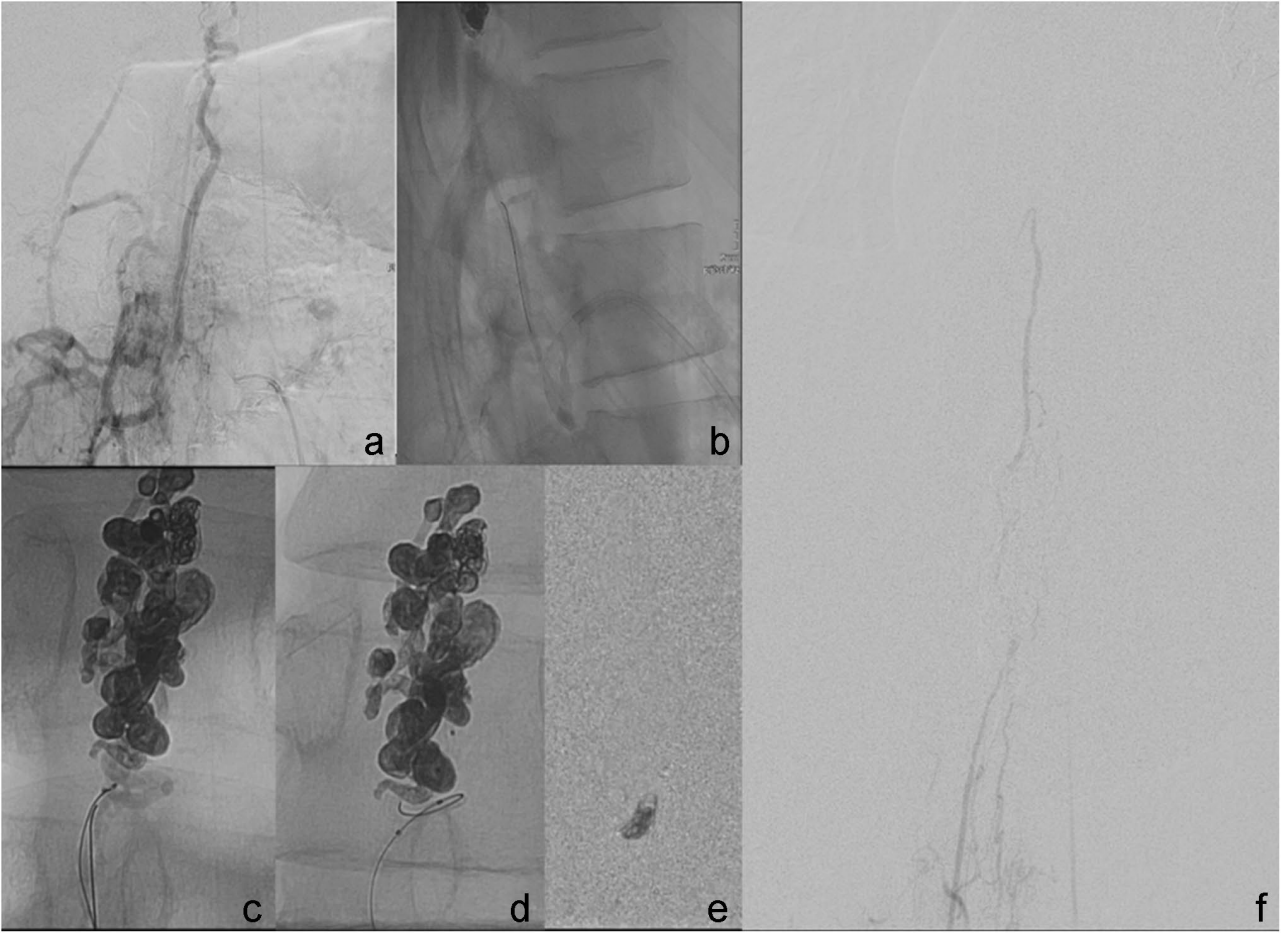
Patient 2. Venous navigation guided by 7F catheter (**a**, **b**) with two microcatheters advanced to the residual AVM nidus via the right intersegmental vein L1 (**c**–**e**). After embolization, injection in L1 right only shows retrograde filling of Th10 right (**f**)

Therefore, we initiated a second angiographic treatment session. Figure 7 shows the remaining arteriovenous shunt from Th10 right (Fig. 5a) and L1 right (Fig. 5b). After embolization, the shunt volume through Th10 right is minimal. The main feeding artery now is L1 right with a radiculopial artery, which divides into tiny branches below the shunt region. The same injection showed contralateral ASA supply. We considered transarterial embolization through these arteries to carry a high risk of ischemic complications. Therefore, we further investigated the course of the AVM draining vein. The vein showed a straight course down to the right intersegmental spinal vein L1 with a direct connection to the inferior vena cava (VCi). The vascular anatomy is sketched in Fig. 6.

With transarterial roadmap injection of lumbar artery L1 right (Fig. 5c, d), retrograde venous navigation was started. The right femoral vein was punctured, and a 7 F sheath was inserted. A curved 7F guiding catheter (Vista Brite Tip IM1, Johnson & Johnson, New Brunswick, NJ, USA) was proceeded coaxially with a long 4F diagnostic catheter and put into the intersegmental vein L1 right. Retrograde navigation with a marathon catheter (Medtronic, New Brunswick, NJ, USA) and a Traxcess wire (Microvention Terumo, Shibuya, Japan) was obtained close to the nidus (Fig. 7a–c). Due to venous tortuosity, further navigation became impossible. Secondly, a Magic microcatheter was navigated along the marathon, which was finally removed and replaced by a Sonic 1.2 25 microcatheter using a 0.007″ microwire (Hybrid, BALT, Montmorency, France), and we decided to embolize from the achieved position with retrograde pressure cooker technique [12].

After the injection of two injectable coils through the Magic microcatheter, embolization with Squid 18 was successfully performed through the Sonic microcatheter (Fig. 7d, e). Due to the tortuous anatomy of the vein, the microcatheter was not retrieved. In the control angiogram through L1 right, retrograde filling of the posterior spinal artery of Th10 right occurred, and no remnants of the AVM were visible (Fig. 7f).

The patient experienced substantial pain relief within the following days and could be discharged 3 days later.

## Discussion

Endovascular treatment of spinal AVMs is a non-standardized approach to treating this rare disease. So far, reported rates of a complete cure are low [3], and transarterial embolization may be hazardous [9, 10] or limited by access route. These are the first reported cases of transvenous treatments of spinal AVMs with liquid embolics (Onyx/Squid) in pressure cooker technique (12].

The transvenous approach to AVMs principally offers three particular advantages: 1. Endovascular treatment of the lesion gets feasible also when there is no suitable arterial access. 2. The risk of arterial ischemia might be lower since no direct arterial embolization takes place. However, a retrograde arterial filling might occur. 3. The treatment of the vein allows a definite cure of the lesion, whereas arterial embolization often results only in partial occlusion of shunts and the primary veins. The first published series of transvenously treated brain AVMs show promising results regarding cure rates and the rate of arterial ischemic complications. In addition, the rates of periprocedural or delayed complications like venous infarctions are low [15]. This might indicate that the transvenous approach and combination with the pressure cooker technique [12] could be a suitable solution for those lesions.

### Anatomical considerations

The usual venous drainage of the spinal cord is largely similar to the arteries. Case #2 illustrates the venous anatomy very similar to the arterial path. Retrograde navigation from the inferior vena cava was feasible with common 7F guiding material. Case #1 showed venous drainage through the posterior fossa, which can occur in AVMs of the cervical spine.

However, in most spinal AVMs, the draining veins have a significant tortuosity, rendering transvenous navigation impossible. In our two illustrative cases, the route of the venous navigation is mainly straight, with only a few serpentine curves close to the AVM nidus. In case #2, the navigation with a marathon catheter and a Traxcess wire appeared too traumatic due to elastic aggravation of the venous curves.

### Embolic agent

The use of ethylenvinylalcohol-polymer agents is well established in the treatment of vascular brain malformations. Despite controllability and efficacy, a major disadvantage is the toxicity of the solvent DMSO and the higher diffusibility [18]. In spinal vascular anatomy, these facts might increase the risk of ischemic or toxic complications due to the small but highly functional vascular anatomy. Therefore, the transvenous approach could also be beneficial to decrease the amount of embolic agent on the arterial side and hence toxic side effects.

## Conclusion

Transvenous treatment of spinal AVMs in combination with retrograde pressure cooker technique was feasible and effective. The main limitation is the accessibility of the venous anatomy. In case of mild tortuosity of the draining veins, this technique can be considered and potentially offers a higher occlusion rate and fewer ischemic or toxic complications.
